# ﻿Phylogenomics, taxonomy and morphological characters of the Microdochiaceae (Xylariales, Sordariomycetes)

**DOI:** 10.3897/mycokeys.106.127355

**Published:** 2024-07-03

**Authors:** Zhao-Xue Zhang, Yu-Xin Shang, Meng-Yuan Zhang, Jin-Jia Zhang, Yun Geng, Ji-Wen Xia, Xiu-Guo Zhang

**Affiliations:** 1 Shandong Provincial Key Laboratory for Biology of Vegetable Diseases and Insect Pests, College of Plant Protection, Shandong Agricultural University, Taian, 271018, China Shandong Agricultural University Taian China; 2 Institute of Crop Germplasm Resources, Shandong Academy of Agricultural Sciences, Jinan, 250100, China Shandong Academy of Agricultural Sciences Jinan China

**Keywords:** Microdochiaceae, multigene phylogeny, new taxa, phylogenomics, taxonomy

## Abstract

Species of the family Microdochiaceae (Xylariales, Sordariomycetes) have been reported from worldwide, and collected from different plant hosts. The proposed new genus and two new species, *viz.*, *Macroidriella***gen. nov.**, *M.bambusae***sp. nov.** and *Microdochiumaustrale***sp. nov.**, are based on multi-locus phylogenies from a combined dataset of ITS rDNA, LSU, RPB2 and TUB2 with morphological characteristics. *Microdochiumsinense* has been collected from diseased leaves of *Phragmitesaustralis* and this is the first report of the fungus on this host plant. Simultaneously, we annotated 10,372 to 11,863 genes, identified 4,909 single-copy orthologous genes, and conducted phylogenomic analysis based on genomic data. A gene family analysis was performed and it will expand the understanding of the evolutionary history and biodiversity of the Microdochiaceae. The detailed descriptions and illustrations of species are provided.

## ﻿Introduction

*Microdochium* Syd. & P. Syd., is the type genus of the family Microdochiaceae Hern.-Restr., Crous & J.Z. Groenew. This was first described by Syd. & P. Syd. (Sydow 1924). The holotype collection of the type species of *Microdochium*, *M.phragmitis* Syd. & P. Syd. was obtained in Germany from the leaves of *Phragmitesaustralis* (Sydow 1924). *Microdochium* species were collected as endophytes, plant pathogens, and saprophytes, and were frequently isolated from different plant hosts (Von Arx 1987; Glynn et al. 2005; Jewell and Hsiang 2013; Mandyam et al. 2013; Hiruma et al. 2018; Liang et al. 2019; Lu et al. 2023; Zhang et al. 2023a). Prior research has indicated that the classification of *Microdochium* within the Amphisphaeriaceae is supported by its morphological similarities (Parkinson et al. 1981; Samuels and Hallett 1983; Von Arx 1984; Jaklitsch and Voglmayr 2012). Hernández‐Restrepo et al. (2016) was proposed that *Idriella* and *Microdochium* may be closely related genera. Their phylogenetic analysis revealed that *Idriella*, *Microdochium*, and *Selenodriella* formed a distinct monophyletic group within the Xylariales. Therefore, Hernández‐Restrepo et al. (2016) established the new family Microdochiaceae to encompass this clade.

Currently, there are approximately 68 species of *Microdochium* listed in the Index Fungorum (2024), with 45 species being accepted. *Microdochium* has a diverse range of hosts that are widely distributed worldwide (Zhang et al. 2017; Crous et al. 2018, 2019, 2021; Marin-Felix et al. 2019; Huang et al. 2020). However, only a few species of *Microdochium* have the capability to cause diseases, primarily impacting grasses and cereals. Zhang et al. (2015) identified *Microdochiumpaspali* Syd. & P. Syd., which was responsible for causing leaf blight on *Paspalumvaginatum* Sw. Liang et al. (2019) identified *Mi.poae* J.M. Liang & Lei Cai, which induced leaf blight disease in turf grasses like *Poapratensis* and *Agrostisstolonifera* L. Stewart et al. (2019) identified *Mi.sorghi* U. Braun, which was responsible for the development of zonate leaf spots and decay on sorghum species. *Mi.albescens* (Thüm.) Hern.-Restr. & Crous was the causative agent of leaf scald and grain discoloration in rice, leading to a global decrease in rice yield (Dirchwolf et al. 2023). *Mi.bolleyi* (R. Sprague) de Hoog & Herm.-Nijh. was cited as the cause of root necrosis and basal rot in creeping bent grass (Hong et al. 2008). In addition to this, some species of *Microdochium* occur as endophytes or saprophytes. Liu et al. (2022) identified three species, *Microdochiummiscanthi*S.B. Liu, X.Y. Liu, Z. Meng & X.G. Zhang, *Mi.sinense*S.B. Liu, X.Y. Liu, Z. Meng & X.G. Zhang, and *Mi.hainanense*S.B. Liu, X.Y. Liu, Z. Meng & X.G. Zhang, isolated from *Miscanthussinensis* Anderss. and *Phragmitesaustralis* (Cav.) Trin. ex Steud in Hainan, China. Zhang et al. (2023a) collected novel species (*Mi.bambusae* J. Zhang, Z.X. Zhang, & Z. Li, *Mi.nannuoshanense* J. Zhang, Z.X. Zhang, & Z. Li, and *Mi.phyllosaprophyticum* J. Zhang, Z.X. Zhang, & Z. Li) from leaves of Bambusaceae plant as a saprobe.

With the advent of the sequencing era, genomics is increasingly being utilized for phylogenetic studies and can offer additional insights into pathogenic mechanisms (Manamgoda et al. 2011; Schoch et al. 2012; Jeewon et al. 2013; David et al. 2016; Mesny et al. 2021; Tsers et al. 2023). However, at present, only the genome information of three species of this taxon (*Microdochium*) can be retrieved from the NCBI database (https://www.ncbi.nlm.nih.gov/, accessed on 30 April 2024). In this study, we explored the species diversity of *Microdochium* and described one new species and one new host record based on the molecular phylogenetic analyses and morphological observations. In addition, we conducted genome and transcriptome sequencing of the new species, aiming to conduct phylogenetic analysis, and gene structure annotation at the genomic level. By comparing and analyzing the obtained data with existing species genome information, we aim to reveal the genetic relationship and functional differences between the new species and other species. This will gain a more comprehensive understanding of the biological characteristics and evolutionary history of the new taxa.

## ﻿Materials and methods

### ﻿Morphological study

During a series of field visits in 2023 in Hainan Province, China, plant specimens with necrotic spots were collected. Even though specimens harbor multiple fungi, we managed to obtain pure colonies through the single spore isolation (Senanayake et al. 2020) and tissue isolation techniques (Zhang et al. 2023a). We retrieved small fragments (5 × 5 mm) from the damaged leaf edges, treated them by immersion in a 75% ethanol solution for 60 s, followed by rinsing in sterile distilled water for 45 s and a 10% sodium hypochlorite solution for 45 s. Subsequently, specimens were rinsed three times in sterile deionized water for 30 s. The processed fragments were then placed on sterile filter paper to remove excess moisture before being transferred onto PDA for incubation at 24 °C for 3 days. The hyphal tips from growing colonies were transferred to fresh PDA plates. Images were captured using a Sony Alpha 6400L digital camera (Sony Group Corporation, Tokyo, Japan) on days 7 and 14. Microscopic examination of the fungal structures was conducted using an Olympus SZ61 stereo microscope and an Olympus BX43 microscope (Olympus Corporation, Tokyo, Japan), along with BioHD-A20c color digital camera (FluoCa Scientific, China, Shanghai) for recording. All fungal strains were preserved in 15% sterilized glycerol at 4 °C, with each strain stored in three 2.0 mL tubes for future studies. Structural measurements were carried out using Digimizer software (v5.6.0), with a minimum of 25 measurements taken for each characteristic such as conidiophores, conidiogenous cells, and conidia. Specimens were deposited in the HSAUP (Herbarium of Plant Pathology, Shandong Agricultural University) and HMAS (Herbarium Mycologicum Academiae Sinicae), while living cultures were stored in the SAUCC (Shandong Agricultural University Culture Collection) for preservation and further research purposes. Taxonomic information of the new taxa was submitted to MycoBank (http://www.mycobank.org).

### ﻿DNA extraction, amplification and sequencing

Fungal DNA was extracted from fresh mycelia grown on PDA using either the CTAB method or a kit method (OGPLF-400, GeneOnBio Corporation, Changchun, China) (Guo et al. 2000; Zhang et al. 2023a). Four gene regions, LSU, ITS, RPB2, and TUB2 were amplified using the primer pairs listed in Suppl. material 1 (Vilgalys et al. 1990; White et al. 1990; Liu et al. 1999; Sung et al. 2007; Jewell et al. 2013). The amplification reaction was conducted in a 25 μL reaction volume, consisting of 12.5 μL 2 × Hieff Canace® Plus PCR Master Mix (Shanghai, China) (with dye) (Yeasen Biotechnology, Cat No. 10154ES03), 0.5 μL each of forward and reverse primer, and 0.5 μL template genomic DNA, with the volume adjusted to 25 μL using distilled deionized water. PCR products were separated and purified using 1% agarose gel and GelRed (TsingKe, Qingdao, China), and UV light was used to visualize the fragments. Gel extraction was performed using a Gel Extraction Kit (Cat: AE0101-C) (Shandong Sparkjade Biotechnology Co., Ltd., Jinan, China). The purified PCR products were subjected to bidirectional sequencing by Biosune Company Limited (Shanghai, China). The raw data (trace data) were analyzed using MEGA v. 7.0 to obtain consistent sequences (Kumar et al. 2016). All sequences generated in this study were deposited in GenBank under the accession numbers provided in Table 1. The abbreviations of the genera names used in our study are as follows: *I.* = *Idriela*; *S.* = *Selenodriella*; *Ma.* = *Macroidriella*; *Mi.* = *Microdochium*.

**Table 1.** GenBank accession number of the taxa used in phylogenetic reconstruction.

Species	Strain no.	GenBank accession number				References
		ITS	LSU	RPB2	TUB2	
*Cryptostromacorticale*	CBS 218.52	HG934112	MH868531	HG934118	HG934104	Vu et al. (2019)
*Idrielalunata*	CBS 204.56*	KP859044	KP858981	–	–	Hernández‐Restrepo et al. (2016)
	CBS 177.57	KP859043	KP858980	–	–	
*I.chlamydospora*	CGMCC 3.20778*	OL897016	OL897058	–	ON569069	Zhang et al. (2023b)
	GZUIFR 21.922	OL897017	OL897059	–	ON569070	
*I.multiformispora*	CGMCC 3.20779*	OL897018	OL897060	ON568988	ON569071	
	GZUIFR 21.924	OL897019	OL897061	ON568989	ON569072	
	GZUIFR 21.925	OL897020	OL897062	ON568990	ON569073	
***Macroidriellabambusae***	SAUCC 6792-1*	**PP716851**	**PP716512**	**PP729053**	**PP729058**	**This study**
	**SAUCC 6792-2**	**PP716852**	**PP716513**	**PP729054**	**PP729059**	
	**SAUCC 6792-5**	**PP716853**	**PP716514**	**PP729055**	**PP729060**	
	**SAUCC 6113-1**	**PP716854**	**PP716515**	**PP729056**	**PP729061**	
	**SAUCC 6113-3**	**PP716855**	**PP716516**	**PP729057**	**PP729062**	
*Microdochiumalbescens*	CBS 243.83	KP858994	KP858930	KP859103	KP859057	Hernández‐Restrepo et al. (2016)
	CBS 291.79	KP858996	KP858932	KP859105	KP859059	
***Mi.australe***	SAUCC 6322-5-1*	**PP695312**	**PP702043**	**PP716780**	**PP716787**	**This study**
	**SAUCC 6151-1**	**PP695313**	**PP702044**	**PP716779**	**PP716788**	
*Mi.bambusae*	SAUCC 1862-1*	OR702567	OR702576	OR715785	PP445175	Zhang et al. (2023a)
	SAUCC 1866-1	OR702568	OR702577	OR715786	PP445176	
*Mi.bolleyi*	CBS 540.92	KP859010	KP858946	KP859119	KP859073	Hernández‐Restrepo et al. (2016)
	CPC 25994	KP859018	KP858954	KP859127	KP859074	
*Mi.chrysanthemoides*	CGMCC 3.17929*	KU746690	KU746736	–	KU746781	Zhang et al. (2017)
*Mi.chrysopogonis*	GDMCC 3.683	MT988022	MT988024	MW002442	MW002441	Lu et al. (2023)
	LNU-196	MT988020	MT988023	MW002445	MW002442	
*Mi.chuxiongense*	YFCC 8794*	OK586161	OK586160	OK584019	OK556901	Tang et al. (2022)
*Mi.citrinidiscum*	CBS 109067*	KP859003	KP858939	KP859112	KP859066	Hernández‐Restrepo et al. (2016)
*Mi.colombiense*	CBS 624.94*	KP858999	KP858935	KP859108	KP859062	
*Mi.dawsoniorum*	BRIP 65649*	MK966337	–	–	–	Crous et al. (2020)
*Mi. ﬁsheri*	CBS 242.90*	KP859015	KP858951	KP859124	KP859078	Hernández‐Restrepo et al. (2016)
*Mi.graminearum*	CGMCC 3.23525*	OP103966	OP104016	OP236027	–	Gao et al. (2022)
	CGMCC 3.23524	OP103965	OP104015	OP236026	–	
*Mi.hainanense*	SAUCC 210782	OM956296	OM959324	OM981154	OM981147	Liu et al. (2022)
	SAUCC 210781*	OM956295	OM959323	OM981153	OM981146	
*Mi.indocalami*	SAUCC 1016*	MT199884	MT199878	MT510550	MT435653	Huang et al. (2020)
*Mi.insulare*	BRIP 75114a	OQ917075	OQ892168	OQ889560		-
*Mi.lycopodinum*	CBS 146.68	KP858993	KP858929	KP859102	KP859056	Hernández-Restrepo et al. (2016)
	CBS 122885*	KP859016	KP858952	KP859125	KP859080	
*Mi.maculosum*	COAD 3358*	Ok966954	Ok966953	OL310501	–	Crous et al. (2021)
*Mi.majus*	CBS 741.79	KP859001	KP858937	KP859110	KP859064	Hernández-Restrepo et al. (2016)
*Mi.miscanthi*	SAUCC 211092*	OM956214	OM957532	OM981148	OM981141	Liu et al. (2022)
	SAUCC 211093	OM956215	OM957533	OM981149	OM981142	
*Mi.musae*	CBS 143499	MH107894	MH107941	–	–	Crous et al. (2018)
	CBS 143500*	MH107895	MH107942	MH108003	–	
*Mi.nannuoshanense*	SAUCC 2450-1*	OR702569	OR702578	OR715787	PP445177	Zhang et al. (2023a)
	SAUCC 2450-3	OR702570	OR702579	OR715788	PP445178	
*Mi.neoqueenslandicum*	CBS 445.95	KP858997	KP858933	KP859106	KP859060	Hernández-Restrepo et al. (2016)
	CBS 108926*	KP859002	KP858938	KP859111	KP859065	
*Mi.nivale*	CBS 116205*	KP859008	KP858944	KP859117	KP859071	
*Mi.nivalevar.majus*	CBS 177.29	MH855031	MH866500	–	–	Vu et al. (2019)
*Mi.nivalevar.nivales*	CBS 288.50	–	MH868135	–	–	
*Mi.novae-zelandiae*	CPC 29376*	LT990655	–	LT990641	LT990608	Marin-Felix et al. (2019)
	CPC 29693	LT990656	–	LT990642	LT990609	
*Mi.paspali*	HK-ML-1371	KJ569509	–	–	KJ569514	Zhang et al. (2015)
	CBS 138620*	KJ569513	–	–	KJ569518	
*Mi.phyllosaprophyticum*	SAUCC 3583-1*	OR702571	OR702580	OR715789	PP445179	Zhang et al. (2023a)
	SAUCC 3583-6	OR702572	OR702581	OR715790	PP445180	
*Mi.phragmitis*	CBS 285.71*	KP859013	KP858949	KP859122	KP859077	Hernández-Restrepo et al. (2016)
	CBS 423.78	KP859012	KP858948	KP859121	KP859076	
*Mi.poae*	CGMCC 3.19170*	MH740898	–	MH740906	MH740914	Liang et al. (2019)
	LC 12115	MH740901	–	MH740909	MH740917	
	LC 12116	MH740902	–	MH740910	MH740918	
*Mi.ratticaudae*	BRIP 68298*	MW481661	MW481666	MW626890	–	Crous et al. (2021)
*Mi.rhopalostylidis*	CBS 145125*	MK442592	MK442532	MK442667	–	Crous et al. (2019)
*Mi.salmonicolor*	NC14-294	MK836110	MK836108	–	–	Das et al. (2020)
*Mi.seminicola*	CBS 139951*	KP859038	KP858974	KP859147	KP859101	Hernández-Restrepo et al. (2016)
	CPC 26001	KP859025	KP858961	KP859134	KP859088	
	DAOM 250161	KP859034	KP858970	KP859143	KP859097	
*Mi.shilinense*	CGMCC 3.23531*	OP103972	OP104022	–	OP242834	Gao et al. (2022)
*Mi.sinense*	SAUCC 211097*	OM956289	OM959225	OM981151	OM981144	Liu et al. (2022)
	SAUCC 211098	OM956290	OM959226	OM981152	OM981145	
	**SAUCC 3922-1**	**PP695314**	**PP702045**	**PP716781**	**PP716789**	**This study**
	**SAUCC 3922-3**	**PP695315**	**PP702046**	**PP716782**	**PP716790**	
*Mi.sorghi*	CBS 691.96	KP859000	KP858936	KP859109	KP859063	Hernández-Restrepo et al. (2016)
*Mi.tainanense*	CBS 269.76*	KP859009	KP858945	KP859118	KP859072	
	CBS 270.76	KP858995	KP858931	KP859104	KP859058	
*Mi.trichocladiopsis*	CBS 623.77*	KP858998	KP858934	KP859107	KP859061	
*Mi.yunnanense*	SAUCC 1011*	MT199881	MT199875	MT510547	MT435650	Huang et al. (2020)
	SAUCC 1012	MT199882	MT199876	MT510548	MT435651	
*Selenodriellacubensis*	CBS 683.96	KP859053	KP858990	–	–	Hernández-Restrepo et al. (2016)
*S.fertilis*	CBS 772.83	KP859055	KP858992	–	–	Hernández-Restrepo et al. (2016)

Notes: Ex-type or ex-epitype strains are marked with “*” and the new species described in this study was marked in bold.

### ﻿Library construction, quality control and whole-genome sequencing

Library construction and sequencing were carried out by Novogene Co., Ltd. (Beijing, China). Obtain FASTQ format data, which included sequence information and corresponding sequencing quality information (Cock et al. 2010). Preprocess the raw data that were obtained from the sequencing platform using fastp (https://github.com/OpenGene/fastp) to obtain clean data for subsequent analysis (Chen et al. 2018). Clean data were deposited in the National Center for Biotechnology Information (NCBI) under BioProject PRJNA1105317.

### ﻿Genome assembly and annotation

Genome data were assembled using the software SPAdes v 3.12.0 (Bankevich et al. 2012). Genome annotation mainly included three aspects: a. Masking of repetitive sequences (RepeatMasker version v4.1.4; RepeatModeler v2.0.3, https://www.repeatmasker.org/); b. Annotation of non-coding RNA (RNAmmer v1.2; tRNAscan-SE v2.0); c. Annotation of gene structure (RNA-seq prediction: Trinity v2.14.0, HISAT2 v2.2.1, StringTie v2.2.0; Ab inito prediction: BRAKER2; Homology protein prediction: GeMoMa v1.9) (Grabherr et al. 2011; Pertea et al. 2015; Keilwagen et al. 2016, 2018; Bruna et al. 2021). The final genome and annotation files were integrated using EVM and PASA (Haas et al. 2008, 2011).

### ﻿Phylogeny

The generated consensus sequences were subjected to Megablast searches to identify closely related sequences in the NCBI’s GenBank nucleotide database (Zhang et al. 2000). Newly generated sequences in this study were aligned with related sequences retrieved from GenBank (Table 1) using MAFFT 7 (Katoh et al. 2019; http://mafft.cbrc.jp/alignment/server/) online service with the default strategy and corrected manually used MEGA 7. For phylogenetic analyses, we operated following the methods by Zhang et al. (2023a), single and concatenated ITS rDNA, LSU, RPB2 and TUB2 sequence alignments were subjected to analysis by maximum likelihood (ML) and Bayesian Inference (BI) algorithms, respectively. ML and BI were run on the CIPRES Science Gateway portal (https://www.phylo.org/, accessed on 30 April 2023) or offline software (ML was operated in RaxML-HPC2 on XSEDE v8.2.12, and BI analysis was operated in MrBayes v3.2.7a with 64 threads on Linux). For ML analyses, the default parameters were used and 1,000 rapid bootstrap replicates were run with the GTR+G+I model of nucleotide evolution; BI analysis was performed using a fast bootstrap algorithm with an automatic stop option (Zhang et al. 2023a). The GTR+I+G model was recommended for LSU, RPB2, and TUB2, while SYM+I+G was suggested for ITS. The Markov chain Monte Carlo (MCMC) analysis of the five concatenated genes was conducted over 1,130,000 generations, yielding 22,602 trees. Following the discard of the initial 5,650 trees generated during the burn-in phase, the remaining trees were used to compute posterior probabilities in the majority rule consensus trees.

For phylogenomic analyses, the genome sequences were submitted to GenBank under the accession numbers in Table 2. The final annotated data were processed to retain the coding protein genes and the longest transcript. Extracted all coding protein genes to identify gene families and single copy orthologous genes using OrthoFinder v2.5.5 (https://github.com/davidemms/OrthoFinder), according to the method by Emms and Kelly (2015, 2019). Multiple sequence alignment was used ParaAT v1.0 (https://ngdc.cncb.ac.cn/tools/paraat) and merged into supergene using seqkit v2.7.0 (https://github.com/shenwei356/seqkit) (Zhang et al. 2012; Shen et al. 2016). Phylogenomic analysis was carried out following the methods by Stamatakis et al. (2014), using RAxML-NG v1.2.1 (https://github.com/amkozlov/raxml-ng) with the LG+G8+F model and 100 bootstrap replications. All resulted trees were plotted using FigTree v. 1.4.4 (http://tree.bio.ed.ac.uk/software/figtree) or ITOL: Interactive Tree of Life (https://itol.embl.de/, accessed on 20 October 2023) (Letunic and Bork 2021) and the layout of the trees was edited in Adobe Illustrator CC 2019.

**Table 2.** BioSample and SRA NCBI number of the taxa used in phylogenomic reconstruction in this study.

Species	Strains	BioSample	SRA NCBI*	References
*Asterophoraparasitica*	AP01	SAMN09737569	SRS3956156	
*Cryphonectriaparasitica*	EP155	SAMN02744051	SRS6915724	Crouch et al. 2020
*Diaportheeres*	CBS 160.32	SAMN21449118	SRS10459569	Hilário et al. 2022
***Macroidriellabambusae***	**SAUCC 6792-1**	**SAMN41099213**	**SRR28834790**	**This study**
***Microdochiumaustrale***	**SAUCC 6322-5-1**	**SAMN41099214**	**SRR28834789**	**This study**
***Mi.bambusae***	**SAUCC 1862-1**	**SAMN41099215**	**SRR28834788**	**This study**
*Mi.bolleyi*	J235TASD1	SAMN04386150	SRS1667728	David et al. 2016
***Mi.nannuoshanense***	**SAUCC 2450-1**	**SAMN41099216**	**SRR28834787**	**This study**
*Mi.nivale*	F00608	SAMN26062287	SRS14642463	Tsers et al. 2023
***Mi.phyllosaprophyticum***	**SAUCC 3583-1**	**SAMN41099217**	**SRR28834786**	**This study**
*Mi.trichocladiopsis*	MPI-CAGE-CH-0230	SAMN06297163	SRS2394902	Mesny et al. 2021
*Pestalotiopsisfici*	W106-1	SAMN02369365		Wang et al. 2015
*Xylariaflabelliformis*	G536	SAMN11912834	SRS4852315	

Species information described in this study is marked in bold.

## ﻿Results

### ﻿Phylogenetic and phylogenomic analyses

**Figure 1.**
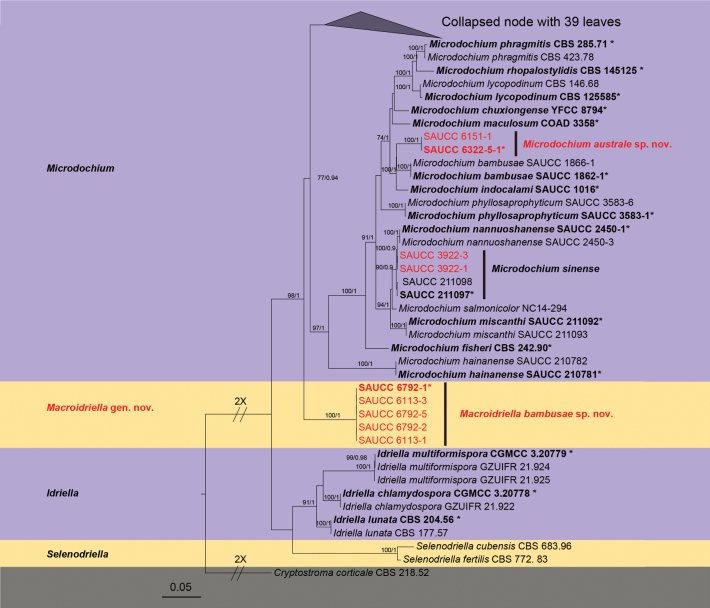
A maximum likelihood tree was constructed using a combined dataset of ITS, LSU, RPB2, and TUB2 sequence data. Branch support values, shown as ML/BIPP, are indicated above the nodes: MLBV ≥ 70% on the left and BIPP ≥ 0.90 on the right. Ex-type cultures are denoted in bold and marked with an asterisk (*). Strains from the current study are highlighted in red. The tree was rooted with *Cryptostromacorticale* (CBS 218.52). The scale bar at the bottom center represents 0.05 substitutions per site.

A total of 80 isolates representing species within the Microdochiaceae family used for phylogenetic analysis. One strain of *Cryptostromacorticale* (CBS 218 52) was used as an outgroup taxon. The final alignment comprised 3,386 concatenated characters, spanning from positions 1 to 553 (ITS), 554 to 1,827 (LSU), 1,828 to 2,676 (RPB2), and 2,677 to 3,386 (TUB2). The maximum likelihood (ML) optimization likelihood was calculated to be -23041.844775. The matrix exhibited 1,071 distinct alignment patterns, with 25.57% of characters or gaps remaining undetermined. MrModelTest suggested that Dirichlet base frequencies be utilized for the ITS, LSU, RPB2, and TUB2 data partitions. The alignment exhibited a total of 876 unique site patterns (ITS: 287, LSU: 186, RPB2: 386, TUB2: 213). The topology of the ML tree corroborated that of the tree obtained from Bayesian inference; therefore, only the ML tree is depicted (Fig. 1). Based on the four-gene phylogeny (Fig. 1), the 80 strains were classified into 47 species. To enhance the visual appeal and conciseness of the phylogenetic tree, 39 strains were collapsed within it (The complete ML phylogenetic tree is available in the Suppl. material 5). Among them, five strains (SAUCC 6792-1, SAUCC 6792-2, SAUCC 6792-5, SAUCC 6113-1 and SAUCC 6113-3) identified a new genus, *Macroidriella* gen. nov., with solid support (98% MLBV and 1.0 BIPP), and *M.bambusae* sp. nov. (SAUCC 6792-1) as the type species. Two strains (SAUCC 6322-5-1 and SAUCC 6151-1) identified as *Microdochiumaustrale* sp. nov.

**Figure 2.**
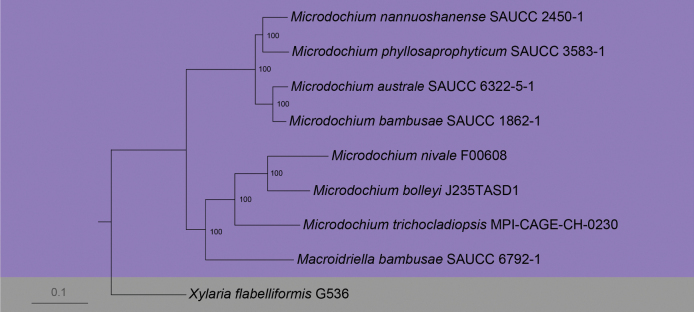
A Maximum Likelihood phylogenomic tree was constructed using a combined 4,909 clusters of orthologous proteins. Maximum Likelihood bootstrap values (≥ 70%) are indicated along branches. Genera are highlighted in different colors. The scale bar at the bottom represents 0.1 substitutions per site.

We sequenced the genomes of six species in Microdochiaceae for phylogenomic analyses, and downloaded the published genomes of four species from in NCBI Datasets (https://www.ncbi.nlm.nih.gov/datasets/). *Xylariaflabelliformis* G536 was used as an outgroup taxon. Based on 4,909 clusters of orthologous proteins, the ML tree is depicted (Fig. 2). The phylogenomic tree was divided into two clades (excepted outgroup), viz, clade 1 (*Microdochiumnannuoshanense* SAUCC 2450-1, *Mi.phyllosaprophyticum* SAUCC 3583-1, *Mi.australe* SAUCC 6322-5-1 and *Mi.bambusae* SAUCC 1862-1) and clade 2 (*Mi.nivale* F00608, *Mi.bolleyi* J235TASD1, *Mi.trichocladiopsis* MPI-CAGE-CH-0230 and *Macroidriellabambusae* SAUCC 6792-1). The branch length of all four strains was < 0.1 in clade 1, indicating that their evolutionary distance was relatively close compared to clade 2 (each strain’s branch was > 0.1). Due to limited genomic data, *Macroidriellabambusae* (SAUCC 6792-1) was not individually clustered, but the evolutionary distance of *Macroidriellabambusae* is relatively far compared to other species.

### ﻿Annotations and comparative analysis

**Figure 3.**
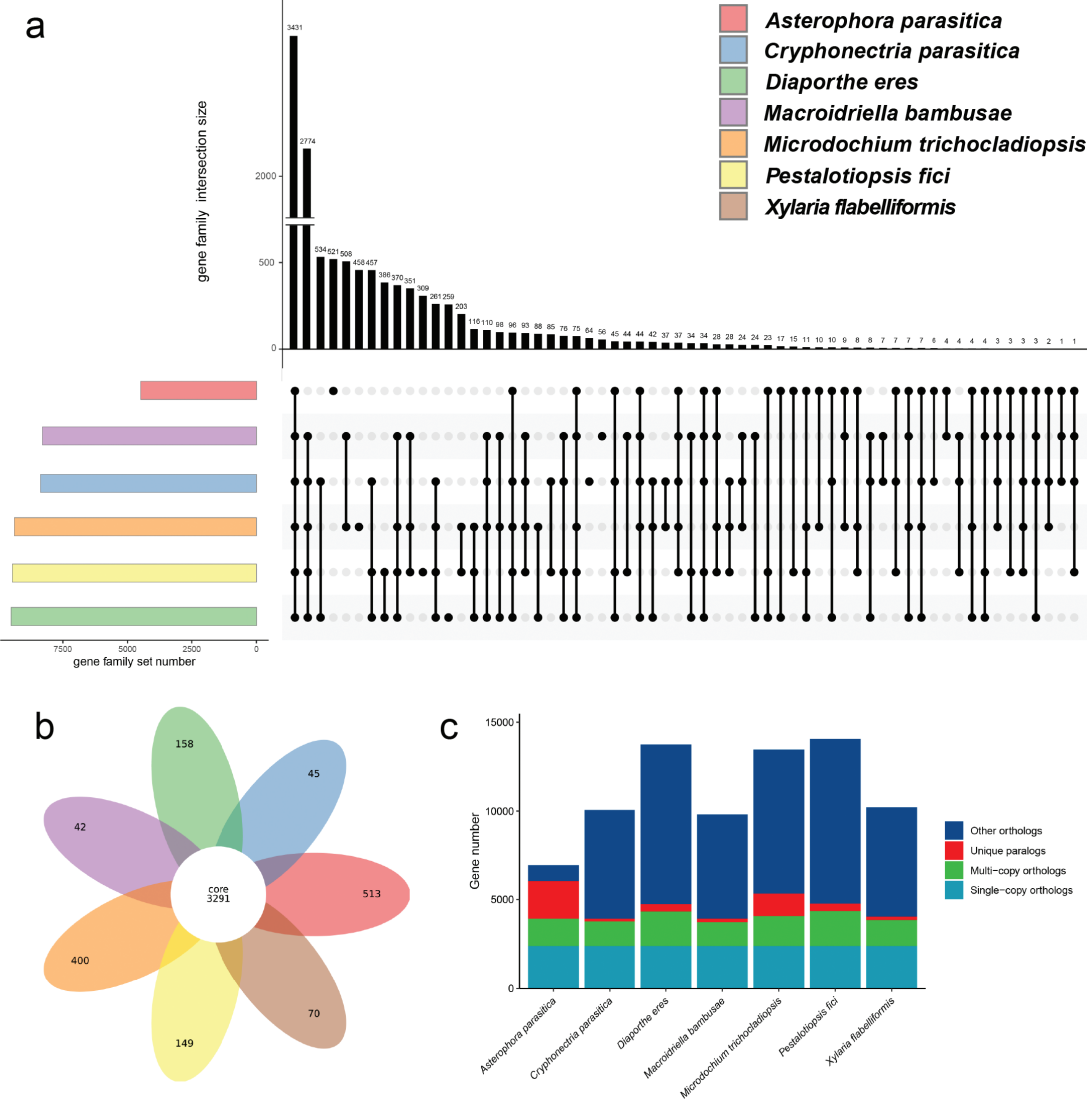
Gene family analysis of *Macroidriella***a** UpSet plot of six strains, showing the intersection counts between different strains in the form of a bar graph **b** petal plot of seven strains, the center of the petal represents the number of shared genes **c** bar chart of homologous genes for each strain.

After structural annotation of the genomic data, we conducted a statistical summary, including, number of genes, total number of cds, total number of exons, total number of introns, total cds length, total exon length and total intron length (Suppl. material 2). Due to the limited genomic data available for Microdochiaceae, we will conduct gene family analysis by comparing the self-tested data of the new genus (*Macroidriella*) with genomic data from the orders of Diaporthales (*Cryphonectriaparasitica* EP155 and *Diaportheeres* CBS 160.32), Xylariales (*Pestalotiopsisfici* W106-1 and *Xylariaflabelliformis* G536), and the Basidiomycota (*Asterophoraparasitica* AP01). The intersections of gene family among the six representative strains (≤ 6) are 3431, the maximum number (508) of gene family intersections between *Macroidriellabambusae* and *Microdochiumtrichocladiopsis*, and the minimum number (4) of gene family intersections between *Macroidriellabambusae* and *Asterophoraparasitica* (Fig. 3a). The intersections of gene family among the seven representative strains are 3,291, the unique number of genes in *Asterophoraparasitica* was 513 (maximum), the unique number of genes in *Macroidriellabambusae* was 42 (minimum) (Fig. 3b). We have presented the number of single-copy genes, multi-copy genes and so on for the seven representative strains (Fig. 3c).

### ﻿Taxonomy

#### ﻿ Macroidriella

Taxon classificationFungi

Z.X. Zhang, J. W. Xia & X.G. Zhang gen. nov.

FBBD2E10-34F1-5D73-AA01-FD415271D1E2

 853699

##### Type species.

*Macroidriellabambusae* Z. X. Zhang, J. W. Xia & X. G. Zhang.

##### Etymology.

Referring to the composed of “Macro-” and “-idriella” (Similar in morphology to *Idriella* and bigger than *Idriella* in conidia).

##### Description.

Genus of Microdochiaceae. ***Endogenic*** on diseased leaves of Bambusaceae sp. ***Sporodochia*** yellowish brown, slimy. ***Conidiophores*** are indistinct and often reduced to conidiogenous cells. ***Conidiogenous cells*** are straight or slightly branched, smooth, curved, mono- or polyblastic, terminal, hyaline, septate, cylindrical and ampulliform. ***Conidia*** are solitary, hyaline, lunate, curved, mooned, multi-guttulate, apex rounded, base usually flattened. Sexual morphs were not observed, chlamydospores were not observed.

##### Notes.

In the phylogenetic tree (Fig. 1), *Macroidriella* is allied to *Idriella*, *Microdochium* and *Selenodriella*, but forms a separate lineage with good statistical support (98% MLBV and 1.0 BIPP). In morphology, the conidia of *Macroidriella* are predominantly lunate and curved, unlike the elliptical conidia of *Microdocium*, suggesting a genus of its own, because it is similar to *Idriella* in morphology (but the conidia of *Macroidriella* are longer than *Idriella*), both being lunate conidia, it is named *Macroidriella* gen. nov.

#### ﻿ Macroidriella bambusae

Taxon classificationFungi

Z.X. Zhang & X.G. Zhang sp. nov.

6E0087DC-C9C4-5D19-A3D2-D51F318C0591

 853712

Fig. 4

##### Type.

China, Hainan Province, Danzhou City: Hainan tropical botanical garden, on diseased leaves of Bambusaceae sp., 15 October 2023, Z. X. Zhang (HMAS 352974, holotype), ex-holotype living culture SAUCC 6792-1.

**Figure 4.**
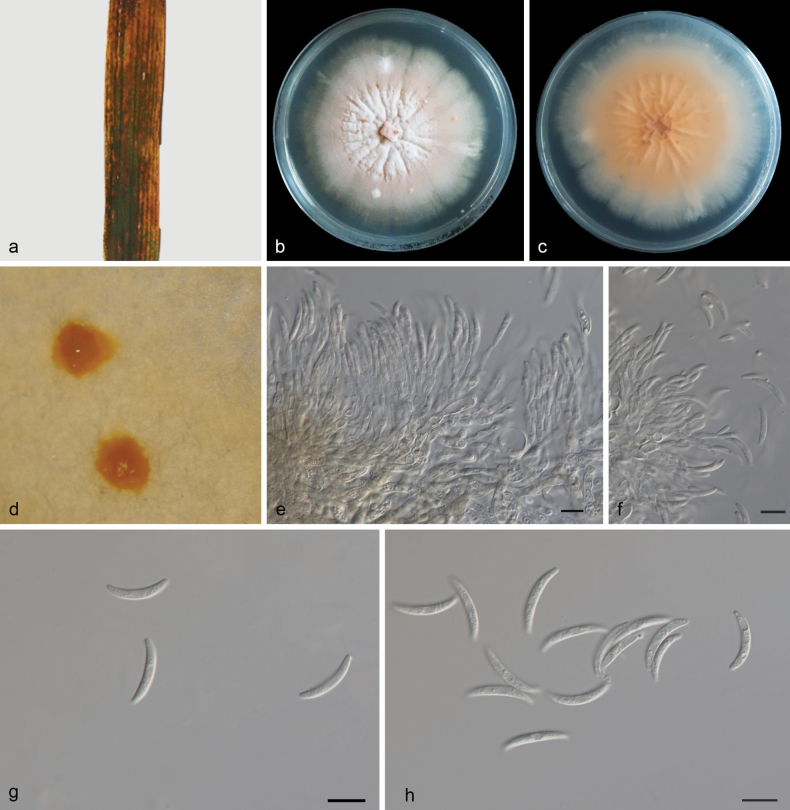
*Macroidriellabambusae* (HMAS 352974, holotype) **a** a leaf of Bambusaceae sp. **b, c** colonies on PDA from above and below after 14 days **d** colony overview **e, f** conidiogenous cells and conidia **g, h** conidia. Scale bars: 10 μm (**e–h**).

##### Etymology.

Referring to the species name of the host plant Bambusaceae sp.

##### Description.

***Endogenic*** on diseased leaves of Bambusaceae sp. ***Mycelia*** are superficial and immersed, 2–3.5 µm wide, branched, membranous and hyaline. ***Sporodochia*** yellowish brown, slimy. ***Conidiophores*** are indistinct and often reduced to conidiogenous cells. Conidiogenous cells are straight or slightly curved, 10.4–15 × 1.7–2.8 µm, mono- or polyblastic, terminal, hyaline, septate, cylindrical and smooth. ***Conidia*** are solitary, hyaline, lunate, curved, mooned, 16.5–21.7 × 2–2.8 µm, multi-guttulate, apex rounded, base usually flattened. Sexual morphs were not observed, chlamydospores were not observed, see Fig. 4.

##### Culture characteristics.

Cultures incubated on PDA at 25 °C in darkness, reaching 63–70 mm diam., had a growth rate of 4.5–5.0 mm/day after 14 days, with moderate aerial mycelia, the center and edges are milky white, with a yellow-brown color in the middle, and sporodochia are observed.

##### Additional material studied.

China, Hainan Province, Danzhou City, Hainan tropical botanical garden, on diseased leaves of Bambusaceae sp., 15 October 2023, Z. X. Zhang (HSAUP 6792-2), living culture SAUCC 6792-2; *ibid*, (HSAUP 6792-5), living culture SAUCC 6792-5; on dead leaves, 15 October 2023, Z. X. Zhang (HSAUP 6113-1), living culture SAUCC 6113-1; *ibid*., (HSAUP 6113-3), living culture SAUCC 6113-3.

##### Notes.

Phylogenetic analyses showed that *Macroidriellabambusae* formed an independent clade (Fig. 1), and closely related to *Idriellamultiformispora* (lunate, curved-shaped conidia) and *Microdochiumbolleyi*. The *Ma.bambusae* was distinguished from *I.multiformispora* (CGMCC 3.20779) by 60/520, 22/1222, 74/848 and 57/710 base-pair differences, from *Mi.bolleyi* (CBS 540.92) by 40/514, 19/765, 138/850 and 51/710 base pairs in ITS, LSU, RPB2 and TUB2 sequences, respectively. Morphologically, *Ma.bambusae* (16.5–21.7 × 2–2.8 µm) longer than *I.multiformispora* (8.5–13.5 × 1.0–2 µm) and *Mi.bolleyi* (5–8.7 × 1.6–2.3 µm) in conidia. Therefore, we describe this fungus as a novel species.

#### ﻿ Microdochium australe

Taxon classificationFungi

Z.X. Zhang, & X.G. Zhang sp. nov.

7293E669-1ADD-5216-9037-0BF31FB4D9E4

 853695

Fig. 5

##### Type.

China, Hainan Province, Jianfengling National Forest Park, on diseased leaves of *Phragmitesaustralis*, 13 October 2023, Z. X. Zhang (HMAS 352973, holotype), ex-holotype culture SAUCC 6322-5-1.

##### Etymology.

Referring to the species name of the host plant *Phragmitesaustralis*.

##### Description.

**Figure 5.**
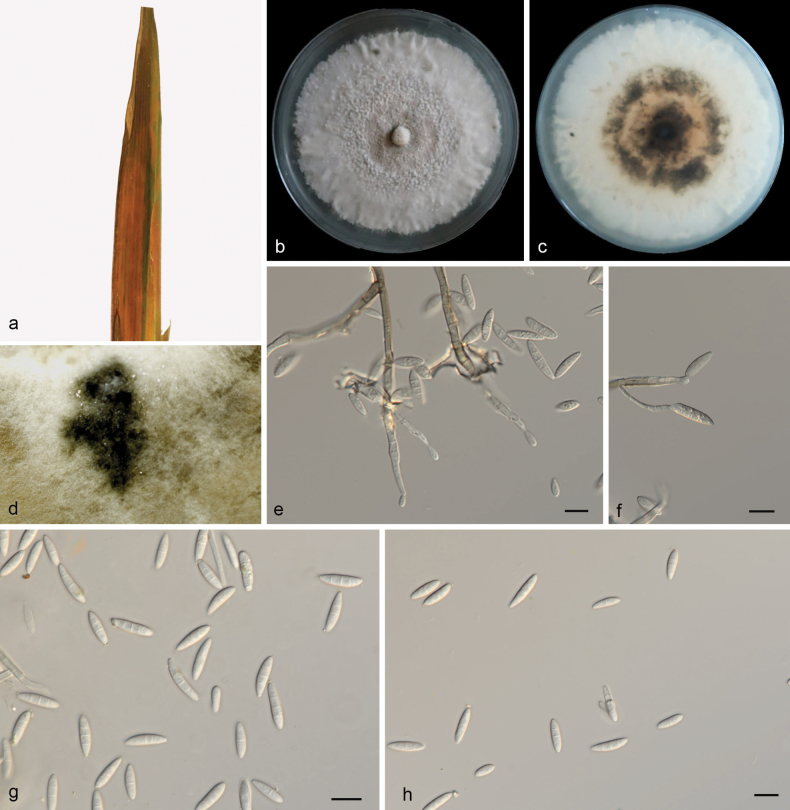
*Microdochiumaustrale* (HMAS 352973, holotype) **a** a leaf of *Phragmitesaustralis***b, c** colonies on PDA from above and below after 14 days **d** colony overview **e, f** conidiogenous cells and conidia **g, h** conidia. Scale bars: 10 μm (**e–h**).

***Endogenic*** on diseased leaves of *Phragmitesaustralis*. ***Mycelia*** are superficial and immersed, 3–3.3 µm wide, branched, membranous and hyaline. ***Sporodochia*** black, aggregative or solitary. ***Conidiophores*** are indistinct and often reduced to conidiogenous cells. ***Conidiogenous cells*** are straight or slightly curved, 15.4–23.5 × 2.8–4 µm, terminal, hyaline, septate, ampulliform or obpyriform, smooth. ***Conidia*** are solitary, hyaline, straight to slight curved, oblong to ellipsoid, 11.3–16.1 × 2.5–3.7 µm, multi-guttulate, (2)3-septate, apex rounded, base usually flattened. Sexual morphs were not observed, chlamydospores were not observed, see Fig. 5.

##### Culture characteristics.

Cultures incubated on PDA at 25 °C in darkness, reaching 73–76 mm diam., had a growth rate of 5.2–5.4 mm/day after 14 days, with moderate aerial mycelia, milky white to grey‐white, with regular margin, and sporodochia are observed, reverses black to brown in the centre, with grey‐white and regular margin.

##### Additional material studied.

China, Hainan Province, Jianfengling National Forest Park, on saprophytic leaves, 13 October 2023, Z. X. Zhang (HSAUP 6151-1), living culture SAUCC 6151-1.

##### Notes.

Phylogenetic analyses showed that *Microdochiumaustrale* sp. nov. formed an independent clade closely related to *Microdochiumbambusae* and *Microdochiumindocalami* (Fig. 1). *Mi.australe* was distinguished from *Mi.bambusae* (SAUCC 1862-1) by 47/503, 2/836, 56/848 and 17/710 base pair differences, from *Mi.bambusae* and *Mi.indocalami* (SAUCC 1016) by 52/503, 2/848, 44/840 and 17/708 base pairs in ITS, LSU, RPB2 and TUB2 sequences, respectively. Morphologically, *Mi.australe* (11.3–16.1 × 2.5–3.7 µm, (2)3-septate) differs from *Mi.bambusae* (13.0–17 × 2.5–3.5 μm, aseptate) and *Mi.indocalami* in conidia (13–15.5 × 3.5–5.5 μm, 3-septate), and, therefore, we described this fungus as a novel species.

#### ﻿ Microdochium sinense

Taxon classificationFungi

S.B. Liu, X.Y. Liu, Z. Meng & X.G. Zhang, J. Fungi 2022, 8, 577.

95F62E33-8978-51CB-85A4-B99BC9487A5B

Fig. 6

##### Material examined.

China, Hainan Province, Jianfengling National Forest Park, on diseased leaves of *Phragmitesaustralis*, 12 April 2023, Z. X. Zhang (HSAUP 3922-1), living culture SAUCC 3922-1; *ibid*., (HSAUP 3922-3), living culture SAUCC 3922-3.

##### Description.

**Figure 6.**
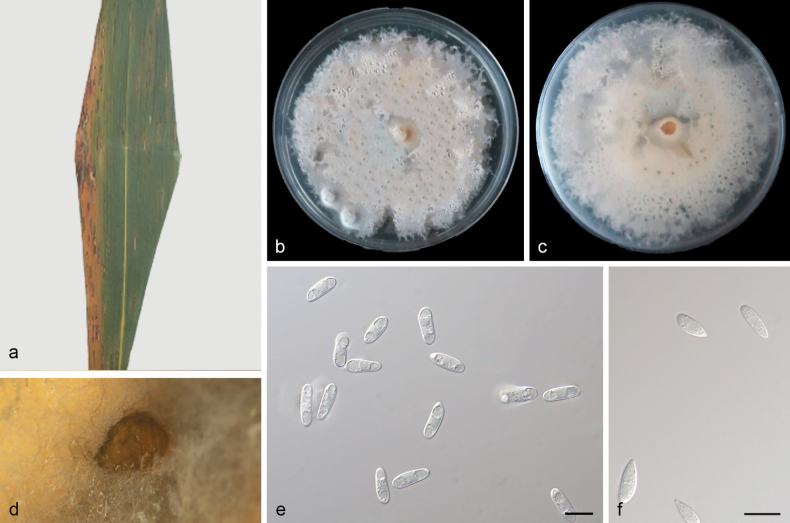
*Microdochiumsinense***a** diseased symptoms on a leaf of *Phragmitesaustralis***b, c** colonies on PDA from above and below after 14 days **d** conidiomata on PDA **e, f** conidia. Scale bars: 10 μm (**e–f**).

***Endogenic*** on diseased leaves of *Phragmitesaustralis*. ***Mycelia*** are superficial and immersed, 2.1–2.9 µm wide, branched, membranous and hyaline. ***Conidia*** are solitary, hyaline, straight, oblong to ellipsoid, 12.3–15 × 3.5–5.6 µm, multi-guttulate, apex rounded, base usually flattened. ***Conidiophores*** were not observed, chlamydospores were not observed, sexual morphs were not observed, see Fig. 6.

##### Culture characteristics.

Cultures incubated on PDA at 25 °C in darkness, reach-ing 72–76 mm diam., had a growth rate of 5.1–5.4 mm/day after 14 days, with moder-ate aerial mycelia, milky white to grey‐white, with irregular margin, reverses light brown in the centre, with grey‐white and regular margin.

##### Notes.

Phylogenetic analyses of four combined genes (ITS, LSU, RPB2 and TUB2) showed that SAUCC 3922-1 and SAUCC 3922-3 clustered with the type collection of *Microdochiumsinense* with strong support (Fig. 1). We, therefore, identified the isolated strains (SAUCC 3922-1 and SAUCC 3922-3) as *Mi.sinense*. Morphologically, the conidia of the both (newly isolated and type) were similar (12.3–15 × 3.5–5.6 vs. 11.5–19.34 × 2.8–5.4 µm).

## ﻿Discussion

The establishment of the family Microdochiaceae by Hernández‐Restrepo et al. (2016) to encompass the clade consisting of *Idriella*, *Microdochium*, and *Selenodriella* within the Xylariales highlights the importance of phylogenetic analysis in understanding the evolutionary relationships among fungi. This new classification helps to better organize and categorize fungal species based on their genetic relatedness and morphological characteristics (Hernández‐Restrepo et al. 2016; Liang et al. 2019; Huang et al. 2020; Liu et al. 2022; Lu et al. 2023; Zhang et al. 2023a). In the recent study, nine strains isolated from two host plants, *Phragmitesaustralis* and Bambusaceae sp., were introduced into a new genus, *Macroidriella* and two new species, *Macroidriellabambusae* and *Microdochiumaustrale*. The Global Biodiversity Information Facility (GBIF) currently hosts 1,594 georeferenced records of Microdochiaceae species worldwide (https://www.gbif.org/, accessed on April 30, 2024). The distribution of this family is predominantly in the United States, Europe, and Oceania, with fewer occurrences in Asia.

In the recent study of the family, *Microdochium* emerged as a prominent research focus, with 12 species of this genus documented across five Provinces (Guizhou, Hainan, Henan, Shandong, and Yunnan) since the beginning of the 21^st^ century in China (Zhang et al. 2015; Liang et al. 2019; Huang et al. 2020; Gao et al. 2022; Liu et al. 2022; Tang et al. 2022). *Microdochium* species have been identified on a variety of host families (10 families), with over half of the fungi associated with Poaceae plants. In contrast, *Idriella* and *Selenodriella* have been less extensively studied, with *Idriella* having only two reported species since the turn of the 21^st^ century. Through the joint analysis of multiple gene fragments and genomes, the position of new taxa can be better determined, especially through phylogenomic analyses, which was provided with more robust support values. Comparative analysis will help us determine the position of the *Macroidriella* genus on the evolutionary tree and its relationship with other fungi. By comparing the genomic data of different fungi, we can identify common gene families and infer their evolutionary relationships. Through comparative genomic analysis, it can be observed that *Macroidriella* has 42 unique single-copy orthologous genes. *Asterophora* shares only 4 single-copy orthologous genes with *Macroidriella*, which also indicates that their relationship is very distant (belonging to different fungal phyla).

This study represents a pioneering effort in Microdochiaceae as it integrates multi-gene fragments with genomic data to unveil the phylogenetic relationships within the family. By combining these diverse datasets, a comprehensive understanding of the evolutionary history of Microdochiaceae is achieved, shedding new light on its genetic landscape and evolutionary dynamics.

## Supplementary Material

XML Treatment for Macroidriella

XML Treatment for Macroidriella bambusae

XML Treatment for Microdochium australe

XML Treatment for Microdochium sinense

## ﻿Additional information

### Author ORCIDs

Zhao-Xue Zhang https://orcid.org/0000-0002-4824-9716

Ji-Wen Xia https://orcid.org/0000-0002-7436-7249

### Data availability

All of the data that support the findings of this study are available in the main text or Supplementary Information.

## Supplementary materials

Supplementary material 1 The PCR primers, sequence and cycles used in this study Data type docx https://binary.pensoft.net/file/1084666This dataset is made available under the Open Database License (http://opendatacommons.org/licenses/odbl/1.0/). The Open Database License (ODbL) is a license agreement intended to allow users to freely share, modify, and use this Dataset while maintaining this same freedom for others, provided that the original source and author(s) are credited.Zhao-Xue Zhang, Yu-Xin Shang, Meng-Yuan Zhang, Yun Geng, Ji-Wen Xia, Xiu-Guo Zhang

Supplementary material 2 GenBank accession number of the taxa used in phylogenetic reconstruction Data type docx https://binary.pensoft.net/file/1084667This dataset is made available under the Open Database License (http://opendatacommons.org/licenses/odbl/1.0/). The Open Database License (ODbL) is a license agreement intended to allow users to freely share, modify, and use this Dataset while maintaining this same freedom for others, provided that the original source and author(s) are credited.Zhao-Xue Zhang, Yu-Xin Shang, Meng-Yuan Zhang, Yun Geng, Ji-Wen Xia, Xiu-Guo Zhang

Supplementary material 3 The sequence of phylogenetic analysis Data type txt https://binary.pensoft.net/file/1084668This dataset is made available under the Open Database License (http://opendatacommons.org/licenses/odbl/1.0/). The Open Database License (ODbL) is a license agreement intended to allow users to freely share, modify, and use this Dataset while maintaining this same freedom for others, provided that the original source and author(s) are credited.Zhao-Xue Zhang, Yu-Xin Shang, Meng-Yuan Zhang, Yun Geng, Ji-Wen Xia, Xiu-Guo Zhang

Supplementary material 4 The sequence of phylogenomic analysis Data type txt https://binary.pensoft.net/file/1084669This dataset is made available under the Open Database License (http://opendatacommons.org/licenses/odbl/1.0/). The Open Database License (ODbL) is a license agreement intended to allow users to freely share, modify, and use this Dataset while maintaining this same freedom for others, provided that the original source and author(s) are credited.Zhao-Xue Zhang, Yu-Xin Shang, Meng-Yuan Zhang, Yun Geng, Ji-Wen Xia, Xiu-Guo Zhang

Supplementary material 5 The complete ML phylogenetic tree Data type pdf https://binary.pensoft.net/file/1084670This dataset is made available under the Open Database License (http://opendatacommons.org/licenses/odbl/1.0/). The Open Database License (ODbL) is a license agreement intended to allow users to freely share, modify, and use this Dataset while maintaining this same freedom for others, provided that the original source and author(s) are credited.Zhao-Xue Zhang, Yu-Xin Shang, Meng-Yuan Zhang, Yun Geng, Ji-Wen Xia, Xiu-Guo Zhang
