# Supplementary material for: ﻿Phylogenomics, taxonomy and morphological characters of the Microdochiaceae (Xylariales, Sordariomycetes)
Source: MycoKeys. 2024 Jul 3;106:303–25. doi: 10.3897/mycokeys.106.127355 (PMC11237568; doi:10.3897/mycokeys.106.127355)
Supplement: Supplementary material 1 — The PCR primers, sequence and cycles used in this study [file mycokeys-106-303-s001.docx]

Table S1. The PCR primers, sequence and cycles used in this study.

| Loci | PCR primers | Sequence (5’ – 3’) | PCR cycles | References |
| --- | --- | --- | --- | --- |
| LSU | LR0R  LR5 | GTA CCC GCT GAA CTT AAG C  TCC TGA GGG AAA CTT CG | (95 °C: 30 s, 51 °C: 60 s, 72 °C: 1 min) × 35 cycles | Vilgalys et al. 1990 |
| ITS | ITS5  ITS4 | GGA AGT AAA AGT CGT AAC AAG G  TCC TCC GCT TAT TGA TAT GC | (95 °C: 30 s, 55 °C: 30 s, 72 °C: 30 s) × 35 cycles | White et al. 1990 |
| RPB2 | RPB2-5F  RPB2-7CR | GAY GAY MGW GAT CAY TTY GG  CCC ATW GCY TGC TTM CCC AT | (95 °C: 30 s, 56 °C: 30 s, 72 °C: 1 min) × 35 cycles | Liu et al. 1999; Sung et al. 2007 |
| TUB2 | Btub526_F  Btub1332_R | CGA GCG YAT GAG YGT YTA CTT  TCA TGT TCT TGG GGT CGA A | (95 °C: 30 s, 56 °C: 30 s, 72 °C: 45 s) × 35 cycles | Jewell et al. 2013 |
