# Supplementary material for: ﻿Phylogenomics, taxonomy and morphological characters of the Microdochiaceae (Xylariales, Sordariomycetes)
Source: MycoKeys. 2024 Jul 3;106:303–25. doi: 10.3897/mycokeys.106.127355 (PMC11237568; doi:10.3897/mycokeys.106.127355)
Supplement: Supplementary material 2 — GenBank accession number of the taxa used in phylogenetic reconstruction [file mycokeys-106-303-s002.docx]

Table S2. GenBank accession number of the taxa used in phylogenetic reconstruction.

| Category | *Ma. bambusae* | *Mi. australe* | *Mi. bambusae* | *Mi. nannuo-shanense* | *Mi. phyllosapro-phyticum* |
| --- | --- | --- | --- | --- | --- |
| Number of genes: | 10,372 | 10,517 | 11,863 | 11,466 | 11,225 |
| Total number of cds: | 27,383 | 25,999 | 28,799 | 27,944 | 27,513 |
| Total cds length: | 16,144,020 | 16,426,143 | 18,314,020 | 17,762,747 | 17,256,408 |
| Total number of exons: | 27,993 | 26,508 | 29,370 | 28,482 | 28,014 |
| Total exon length: | 20,809,754 | 19,937,750 | 22,532,449 | 21,780,047 | 21,365,459 |
| Total number of introns: | 17,621 | 15,991 | 17,507 | 17,016 | 16,789 |
| Total intron length: | 1,911,361 | 1,564,904 | 1,706,186 | 1,727,341 | 1,727,974 |
