# Supplementary figures and images for: ﻿Phylogenomics, taxonomy and morphological characters of the Microdochiaceae (Xylariales, Sordariomycetes)
Source: MycoKeys. 2024 Jul 3;106:303–25. doi: 10.3897/mycokeys.106.127355 (PMC11237568; doi:10.3897/mycokeys.106.127355)

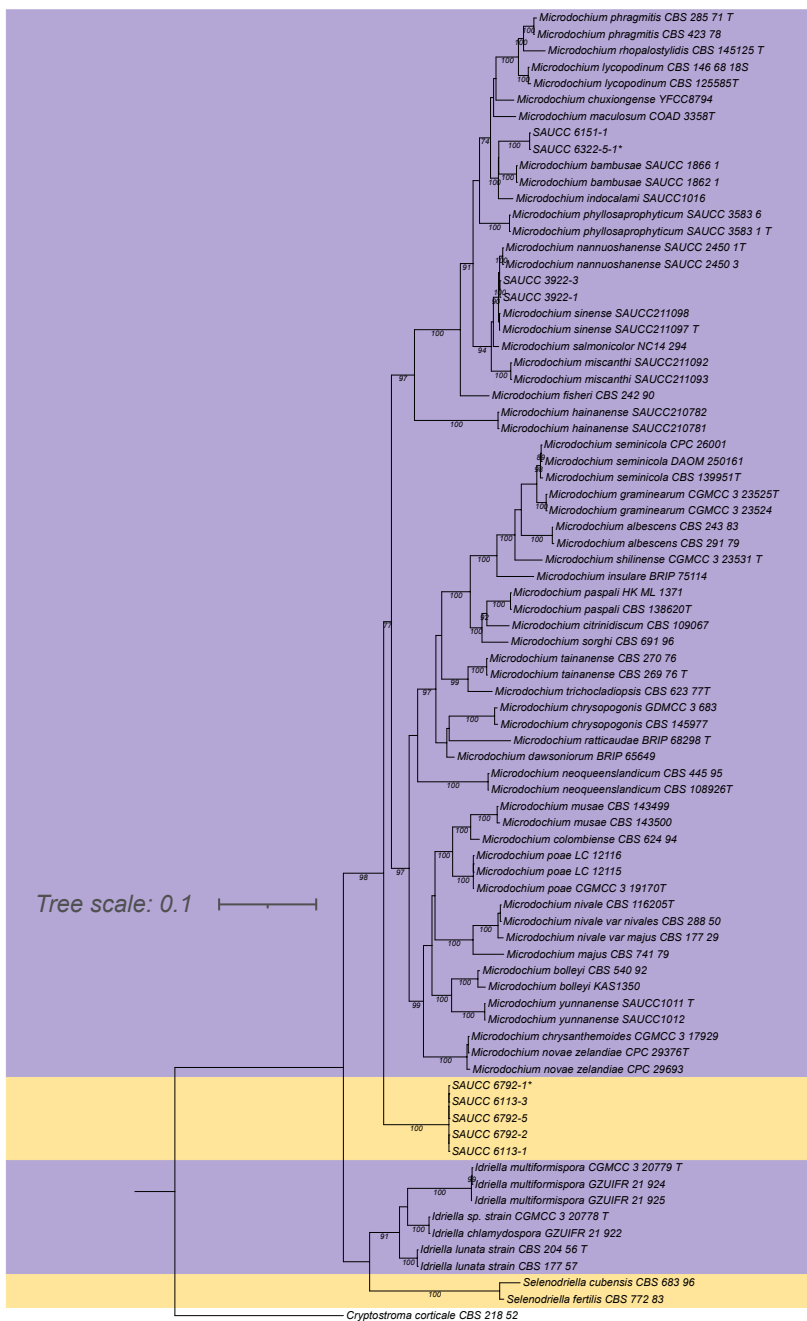

Supplement: Supplementary material 5 — The complete ML phylogenetic tree [file mycokeys-106-303-s005.pdf]
